# 

**Published:** 1871-09

**Authors:** 


### CONTENTS:

ORIGINAL COMMUNICATIONS:

ARTICLE I. Water: Free Heat and Water as Remedial Agents. By T. Curtis Smith, M.D. .......... 513

ART. II. Statistical Report on Cataract. By E. L. Holmes M.D. Read at the meeting of the Illinois Med. Society held at Peoria, May, 1871.. 519

ART. III. Laceration of Perineum and Rectum. By Irael B. Washburn, M.D. .......... 525

ART. IV. Foreign Body in Nose—Failure in Diagnosis—Spontaneous Recovery. By C. T. Fenn, M.D... 526

ART. V. Gynecological Clinique. N. Chicago Charity Dispensary, Rush Med. College. Reported by C. T. Fenn, M.D. .......... 527

ART. VI. Cholera, and its Relation to Pregnancy and Child-Birth. By Carl Proegler, M.D. .......... 536

HOME CORRESPONDENCE .......... 542

SELECTIONS:

On Syphilitic Epilepsy. By R. A. Vance, M.D. .......... 549

EDITOR'S BOOK TABLE .......... 559

EDITORIAL .......... 566

PROCEEDINGS OF SOCIETIES .......... 570

